# Repetition Suppression for Speech Processing in the Associative Occipital and Parietal Cortex of Congenitally Blind Adults

**DOI:** 10.1371/journal.pone.0064553

**Published:** 2013-05-22

**Authors:** Laureline Arnaud, Marc Sato, Lucie Ménard, Vincent L. Gracco

**Affiliations:** 1 Centre for Research on Brain, Language and Music, McGill University, Montréal, Canada; 2 Centre for Research on Brain, Language and Music, and GIPSA-lab, Centre national de la recherche scientifique and Grenoble Université, Grenoble, France; 3 Département de Linguistique, Université du Québec à Montréal, Montréal, Canada; 4 School of Communication Sciences and Disorders, McGill University, Montréal, Canada; 5 Haskins Laboratories, New Haven, Connecticut, United States of America; University of Tokyo, Japan

## Abstract

In the congenitally blind (CB), sensory deprivation results in cross-modal plasticity, with visual cortical activity observed for various auditory tasks. This reorganization has been associated with enhanced auditory abilities and the recruitment of visual brain areas during sound and language processing. The questions we addressed are whether visual cortical activity might also be observed in CB during passive listening to auditory speech and whether cross-modal plasticity is associated with adaptive differences in neuronal populations compared to sighted individuals (SI). We focused on the neural substrate of vowel processing in CB and SI adults using a repetition suppression (RS) paradigm. RS has been associated with enhanced or accelerated neural processing efficiency and synchronous activity between interacting brain regions. We evaluated whether cortical areas in CB were sensitive to RS during repeated vowel processing and whether there were differences across the two groups. In accordance with previous studies, both groups displayed a RS effect in the posterior temporal cortex. In the blind, however, additional occipital, temporal and parietal cortical regions were associated with predictive processing of repeated vowel sounds. The findings suggest a more expanded role for cross-modal compensatory effects in blind persons during sound and speech processing and a functional transfer of specific adaptive properties across neural regions as a consequence of sensory deprivation at birth.

## Introduction

In the congenitally blind (CB), numerous neuroimaging studies have demonstrated visual cortical activation to a wide range of sensory processing tasks including auditory change detection [1], spatial sound localization and discrimination [2]–[3], spoken language processing [4]–[5] and Braille reading [6]. The functional nature of cross-modal activation of visual cortex in the blind comes from three different but related sources. Studies using transcranial magnetic stimulation of the visual cortex have demonstrated a causal link between occipital cortex activation and language tasks [7]–[8]. Studies of language processing have demonstrated graded activation patterns in response to increasing processing complexity [4], [6], [9] and behavioural results have yielded evidence of enhanced performance in tasks involving dichotic listening and attention [10], pitch detection [11], auditory localization [12], and speech perception [13]–[17]. From these results, although speculative, enhanced performance relative to sighted controls might be partly linked to cross-modal differences in the CB and early blind (EB) compared to SI. One of the issues not previously addressed in studies of cross-modal plasticity difference is whether visual activity might also be recruited in the CB using passive auditory speech listening and whether cross-modal plasticity in the CB is associated with enhanced or expanded adaptive properties of the neuronal populations associated with the expanded activation. To this aim, we used a repetition suppression (RS) paradigm to identify the neural substrate associated with passive speech listening to repeated vowels in CB and SI adults. Repetition suppression, the attenuation of neural response to repeated stimuli, has been observed in single-unit recordings in non-human primates [18] and in functional neuroimaging studies in humans [19]. Repetition suppression is associated with response priming and is used as a metric to examine the processing characteristic of neuronal populations [20]–[22]. Recent data [23] and theory [24] suggest that RS reflects a combination of attention and predictive mechanisms (predictive coding) integrating top-down expectations with bottom-up sensory input [25]. While a number of theoretical models have been proposed to explain RS [20], [22], [26]–[27] all are associated with increased processing and information encoding efficiencies related to repeated stimulus properties. Here we were interested to what extent within and across-modal activation to passive vowel processing would result in RS effects in the CB. Two groups of ten congenitally blind and ten sighted adults participated in a functional magnetic resonance imaging (fMRI) study. A sparse sampling acquisition technique was used where participants passively listened to single vowel repetitions during the silent interval between successive image acquisitions. While both groups demonstrated RS effects to the passive vowel presentations in the temporal cortex, extended RS effects were observed in visual and parietal cortical regions in the CB. Together with the enhanced performance for sound processing reported in the literature, it appears that the expansion of cortical representation for speech and increased processing efficiency within those recruited cortical areas, may be a hallmark of functional cross-modal reorganization in the CB.

## Materials and Methods

### Participants

Ten congenitally blind participants (4 females, mean age = 39 years, age range = 20–59 years) and ten sighted healthy adults (5 females, mean age = 35 years, age range = 22–59 years) comprised the experimental group. There was no significant difference in age between the two groups (t-value = 0.356, p = 0.726). The CB participants had complete congenital visual impairment, classified as category 5 (no light perception) except for one participant who was classified as category 4 (light perception) presenting distance visual acuity worse than 20/1200. The cause of blindness was not obtained. However during the recruitment process all blind participants declared that they were never able to see.

All participants were native Canadian French speakers, right-handed with no history of speech or hearing disorders. The experiment was performed in accordance with the ethical standards in the 1964 Declaration of Helsinki and requirements of the Faculty of Medicine, McGill University. The experimental and consent procedures were approved by the Research Ethics Board of the Montreal Neurological Institute. All sighted subjects provided written consent. Blind subjects were presented with a Braille copy of the consent form and after reading gave verbal consent.

### Stimuli

The stimuli were multiple /i/ and /y/ French vowels recorded from a native French Canadian male speaker in a sound-attenuated room. Multiple utterances of /i/ and /y/ French vowels were individually recorded. Seven clearly articulated tokens of each vowel were selected and digitized at a sampling rate of 44.1 kHz with 16-bit quantization recording. Using Praat software (Institute of Phonetic Sciences, University of Amsterdam, NL), the fundamental frequency (F_0_), and first, second, and third formant frequencies (F_1_, F_2_, F_3_) values were calculated for each vowel from a section of the vowel located at ±25 ms of the maximum peak intensity. For the /i/ vowels, the mean F_0_, F_1_, F_2_, F_3_, peak intensity and duration values were 155 Hz (±8), 299 Hz (±9), 2287 Hz (±56), 3166 Hz (±35), 70 dB (±2) and 357 ms (±49), respectively. For /y/ vowels, the mean F_0_, F_1_, F_2_, F_3_, intensity and duration values were 156 Hz (±8), 301 Hz (±7), 2061 Hz (±61), 2982 Hz (±103), 73 dB (±2) and 322 ms (±52), respectively.

### Procedure

**Figure 1:**
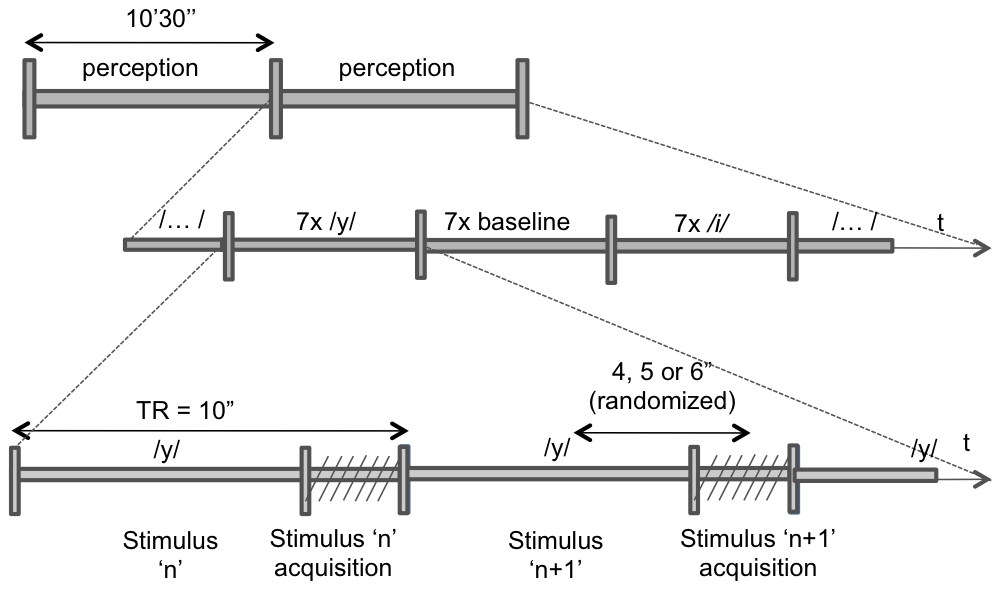
Schematic of the experimental runs. Each run lasted 10.5 minutes and included 63 trials per run (TR = 10 secs; 7 seconds of silence). The /y/ and /i/ vowels, or rest were presented in three sets of 7 consecutive presentations (one vowel or rest per TR repeated 7 times), e.g., 3 repetitions of the sequence—( i i i i i i i y y y y y y y baseline baseline baseline baseline baseline baseline baseline).

The fMRI experiment consisted of two functional runs (63 trials per run) in which participants passively listened to French steady-state vowels (/i/ and /y/). A sparse sampling acquisition paradigm was used (e.g., [28]–[29]) with the speech stimuli or the resting condition presented in the silent interval (7 sec) between volume acquisitions. In each run, the same vowel (/i/ or /y/), or the resting condition was presented in three sets of seven consecutive trials (see Figure 1 for details). This procedure allowed measuring changes in BOLD signal for repeated vowel processing. Blind and sighted participants were instructed to close their eyes, to pay attention to the auditory stimuli and not to move during the experimental session.

### Data acquisition

Magnetic resonance images were acquired with a 1.5T whole-body MRI scanner (Siemens Sonata MR scanner) and standard headcoil in the Brain Imaging Centre (BIC) at the Montreal Neurological Institute. Auditory stimuli were amplified (Rolls RA53b Headphone Amplifier) and presented to participants with MRI compatible insert earphones (Sensimetrics S14) at a comfortable sound pressure level.

Functional images were obtained using a T2*-weighted, echoplanar imaging (EPI) sequence with whole-brain coverage (TR = 10 s, acquisition time = 3000 ms, TE = 51 ms, flip angle = 90°). Each functional scan comprised thirty-five axial slices parallel to the anteroposterior commissural plane acquired in interleaved order (64×64 matrix; field of view: 256 mm^2^; 4×4 mm^2^ in plane resolution with a slice thickness of 4 mm without gap). A high-resolution T1-weighted whole-brain structural image was acquired for each participant after the second functional run (256×256 matrix; field of view: 256 mm^2^; sagittal volume of 256×256×176 mm^3^ with a 1 mm isotropic resolution, TR/TE = 22/9.2 ms with 30% partial echo, flip angle = 30°).

In each functional run and for each TR, the timing between the vowel onset and the midpoint of the following functional scan acquisition was randomly varied between 4 s, 5 s or 6 s. Each functional run was 10.5 minutes in length.

### Data analyses

Data were analysed using the SPM5 software package (Wellcome Department of Imaging Neuroscience, Institute of Neurology, London, UK) running on Matlab (Mathworks, Natick, MA, USA). The maximum activation peaks for each cluster were labelled according to probabilistic cytoarchitectonic maps in the SPM Anatomy toolbox [30]. If a brain region was assigned a probability less than 50% or unspecified in the toolbox, the peak coordinates were converted from MNI space to Talairach space and the brain region identified with the Talairach Daemon [31].

The functional series was realigned for head movement. After segmentation of the T1 structural image and coregistration to the mean functional image, all functional images were spatially normalized into standard stereotaxic space of the Montreal Neurological Institute. All functional images were smoothed using an 8 mm FWHM Gaussian kernel.

A General Linear Model was used to analyse BOLD activity with regressors of interest related to the seven vowel repetitions and six realignment parameters with the silent trials forming an implicit baseline. The BOLD response for each event was modelled using a single-bin finite impulse response (FIR) basis function spanning the time of acquisition (3 s). Before estimation, high-pass filtering (cutoff of 128 s) was applied. Beta weights associated with the modelled FIR responses were then computed to fit the observed BOLD signal time course in each voxel for each condition. Individual statistical maps were calculated for each vowel repetition with the related baseline and subsequently used for group statistics.

A second-level random effect group analysis was carried-out. A mixed analysis of variance (ANOVA) was performed, with the group (2 levels: blind and sighted participants) as a between-subject factor and the vowel repetition (7 levels: R1 to R7) as a within-subject factor.

First, two t-contrasts were calculated to determine brain activity averaged across the seven vowel repetitions (i.e., irrespective of the RS) compared to the resting condition (mean effect of vowel perception: blind>rest and sighted>rest; false discovery rate corrected cluster and voxel levels of p<.001 and cluster extent of at least 30 voxels). To identify specific activity differences between the two groups, two t-contrasts were then calculated (main effect of group: blind>sighted participants and sighted>blind participants; corrected level of p≤.01 at the cluster level and uncorrected level of p<.001 at the voxel level, cluster extent of at least 30 voxels).

Second, in order to identify brain regions showing RS for repeated vowel processing, two t-contrasts were assessed to determine brain regions that showed a significant linear decrease in activity across the 7 vowel repetitions (RS effect: blind and sighted participants; corrected level of p≤.01 at the cluster level and uncorrected level of p<.001 at the voxel level, cluster extent of at least 30 voxels). Exclusive masking was used to identify voxels for which RS effects were not shared between the two groups. The SPM constituting the exclusive mask was thresholded at p<.05, whereas the contrast to be masked was thresholded at an uncorrected level of p<.001 at the voxel level but at a corrected level of p≤.01 at the cluster level and cluster extent of at least 30 voxels).

## Results

### Mean effect of vowel processing

**Figure 2:**
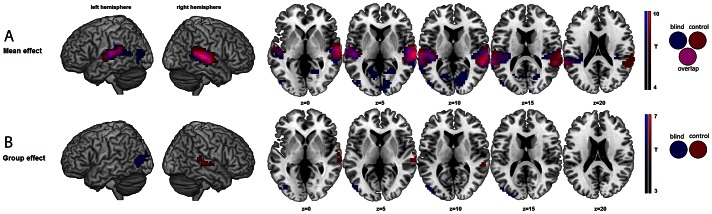
Surface rendering of brain activity for vowel processing. 2A-Surface rendering of brain activity for the **Mean effect** of vowel processing for the blind (blue) and sighted (red) participants compared to rest (Mean effect: false discovery rate corrected level of p<.001 and cluster extent of at least 30 voxels); horizontal sections showing areas of activation from z = 0 to z = 20 in 5 mm increments. 2B-Surface rendering of brain activity for the **Main effect** of group (Group effect: corrected level of p≤.01 at the cluster level and uncorrected level of p<.001 at the voxel and cluster levels, cluster extent of at least 30 voxels); horizontal sections showing areas of activation from z = 0 to z = 20 in 5 mm increments.

Surface rendering of brain activity and maximum activation peaks of the mean effect of vowel processing (compared to the resting condition) for the blind and sighted participants are provided in Figure 2A and Tables 1 & 2. For both blind and sighted participants, auditory vowel processing induced large bilateral activation of the auditory cortex, including activity in the transverse temporal gyrus (primary/secondary auditory cortex) and in the posterior part of the superior temporal gyrus/sulcus. For blind participants, additional bilateral occipital activation was observed in the extrastriate visual cortex with maximum activation peaks located in the left middle occipital gyrus, in the right lingual and parahippocampal gyri and in the cuneus, bilaterally.

**Table 1:** Mean effect of vowel processing compared to rest for blind participants (coordinates in MNI space).

Cluster	Voxels	Regions	BA	H	x	y	z	T
1	250	transverse temporal gyrus	41	L	−52	−24	4	7.80
		transverse temporal gyrus	41	L	−40	−36	12	5.99
		posterior superior temporal gyrus	22	L	−64	−36	12	5.70
2	235	transverse temporal gyrus	41	R	56	−20	4	8.19
		posterior superior temporal gyrus	22	R	56	−36	4	6.72
		posterior superior temporal gyrus	22	R	56	−44	12	6.34
3	126	extrastriate cortex/cuneus	17	R	20	−68	12	6.16
		parahippocampal gyrus	30	R	24	−52	8	5.17
		extrastriate cortex/lingual gyrus	17	R	16	−56	0	5.00
4	47	extrastriate cortex/cuneus	17	L	−12	−68	8	5.60
5	32	extrastriate cortex/middle occipital gyrus	19	L	−48	−80	16	5.10
		extrastriate cortex/middle occipital gyrus	19	L	−48	−80	0	4.95
		extrastriate cortex/middle occipital gyrus	19	L	−40	−76	−4	4.64

**Table 2:** Mean effect of vowel processing compared to rest for sighted participants (coordinates in MNI space).

Cluster	Voxels	Regions	BA	H	x	y	z	T
1	306	posterior superior temporal gyrus	22	R	64	−28	4	10.14
		posterior superior temporal gyrus	22	R	64	−16	0	8.87
2	208	transverse temporal gyrus	42	L	−60	−24	12	8.08
		transverse temporal gyrus	42	L	−64	−32	12	7.77
		posterior superior temporal gyrus	22	L	−64	−40	16	7.47

### Main effect of group

Surface rendering of brain activity and maximum activation peaks of the main effect of group (blind vs. sighted participants) are provided in Figure 2B and Tables 3 & 4. The main effect of group revealed significant activation differences between blind and sighted participants during auditory vowel processing, with stronger neural responses for sighted participants in the right transverse and posterior superior temporal gyri as well as specific activity of the left extrastriate cortex (cuneus and middle occipital gyrus) for blind participants.

**Table 3:** Main effect of vowel processing – blind>sighted participants (coordinates in MNI space).

Cluster	Voxels	Regions	BA	H	x	y	z	T
1	40	extrastriate cortex/middle occipital gyrus	39	L	−52	−76	8	4.11
		extrastriate cortex/middle occipital gyrus	19	L	−44	−84	12	3.93
		extrastriate cortex/middle occipital gyrus	18	L	−24	−96	16	3.50
		extrastriate cortex/middle occipital gyrus	19	L	−40	−76	0	3.44

**Table 4:** Main effect of vowel processing – sighted>blind participants (coordinates in MNI space).

Cluster	Voxels	Regions	BA	H	x	y	z	T
1	31	posterior superior temporal gyrus	22	R	64	−28	4	4.79
		posterior superior temporal gyrus	22	R	60	−8	0	3.42
		transverse temporal gyrus	41	R	48	−36	8	3.28

### Repetition suppression effect

**Figure 3:**
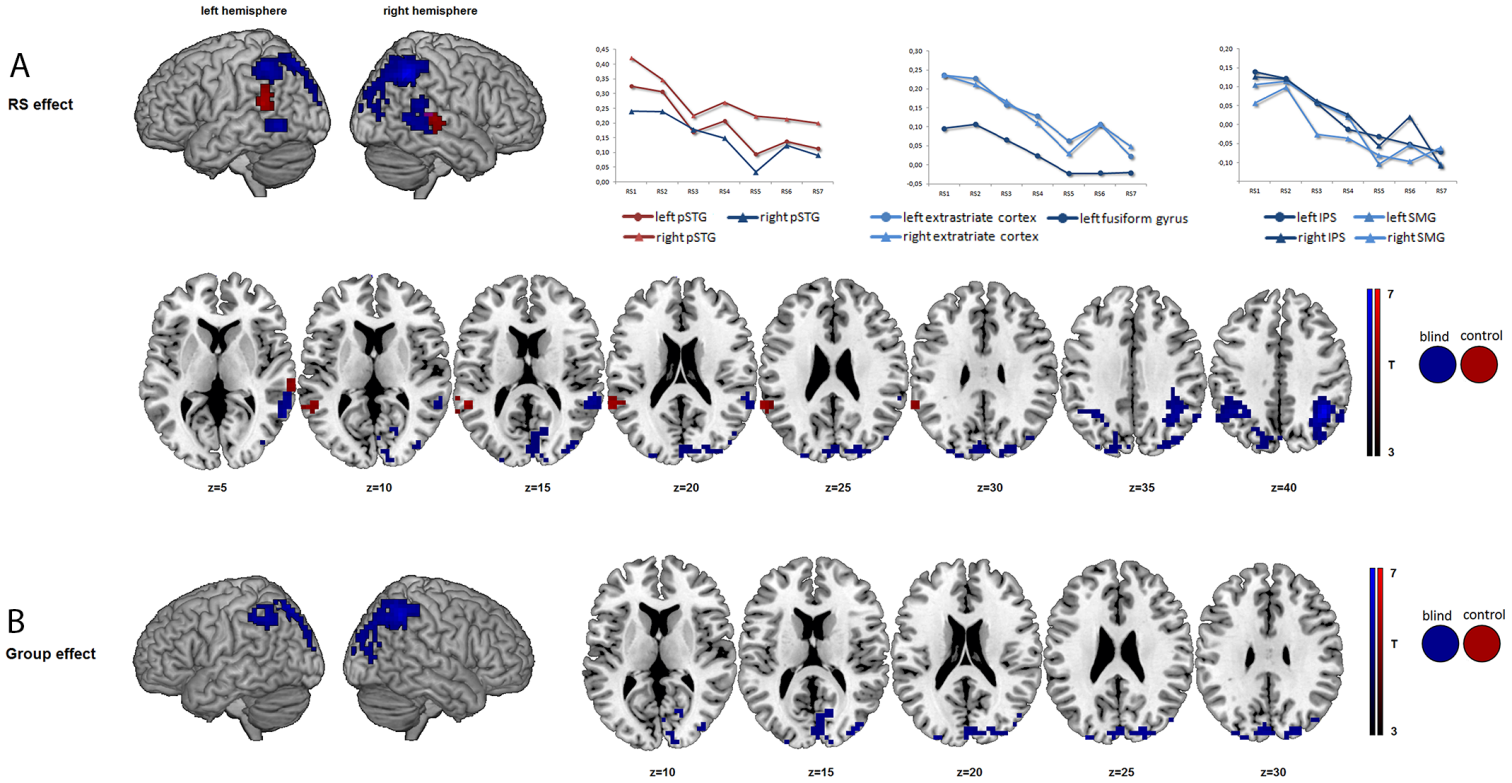
Surface rendering brain activity for the repetition suppression effect and related contrast estimates. 3A-Surface rendering of brain activity for the **Repetition Suppression effect** and related contrast estimates reflecting percentage BOLD signal decrease for the seven vowel repetitions in auditory, visual and parietal regions (RS effect: corrected level of p≤.01 at the cluster level and uncorrected level of p<.001 at the voxel level, cluster extent of at least 30 voxels); 3B-Surface rendering of the Group effect and horizontal sections showing areas of activation for 4 slices from z = 10 to z = 30 in 5 mm increments. Abbreviations: pSTG (posterior superior temporal gyrus); IPS (inferior parietal sulcus); SMG (supramarginal gyrus).

Surface rendering of brain activity and maximum activation peaks of the RS effect for the blind and sighted participants are provided in Figure 3A and Tables 5 & 6. As expected, RS was observed in the auditory cortex during repeated vowel processing. For sighted participants, BOLD decrease across the 7 consecutive vowels was observed bilaterally in the posterior part of the superior temporal gyrus/sulcus and in the right posterior part of the middle temporal gyrus. For blind participants, RS was also observed in the right posterior part of the superior and middle temporal gyri, with RS activity extending dorsally to the ventral part of suparmarginal gyrus. Although no RS was observed in the left posterior superior temporal gyrus for blind participants with an extend threshold of 30 voxels, it should be noted that this region appears sensitive to RS with a lower threshold of 10 voxels (p<.001 uncorrected at the voxel level but not surviving a corrected threshold at the cluster level). Additional RS was observed for blind participants in the left fusiform gyrus and in the bilateral extrastriate visual cortex (with maximum activation peaks located in the cuneus), with RS activity extending in the supramarginal gyrus, the intraparietal sulcus and the superior parietal lobule.

**Table 5:** RS effect for blind participants (coordinates in MNI space).

Cluster	Voxels	Region	BA	H	x	y	z	T	R1	R2	R3	R4	R5	R6	R7
1	637	anterior intraparietal sulcus	40	R	40	−52	44	6.75	0.14	0.12	0.06	−0.01	−0.03	−0.05	−0.07
		anterior intraparietal sulcus	40	L	−44	−48	48	4.86	0.13	0.12	0.06	0.03	−0.06	0.02	−0.11
		superior parietal cortex	7	L	−16	−76	52	4.77	0.11	0.17	0.11	−0.01	−0.09	−0.10	−0.12
2	74	supramarginal gyrus	40	R	60	−40	16	4.36	0.22	0.19	0.11	0.09	0.00	0.07	0.07
		posterior middle temporal gyrus	21	R	60	−52	4	4.26	0.20	0.17	0.14	0.08	−0.02	0.06	0.01
		posterior middle temporal gyrus	21	R	64	−36	−4	4.01	0.17	0.19	0.08	0.12	−0.04	0.03	0.03
3	38	fusiform gyrus	37	L	−52	−60	−4	4.65	0.10	0.11	0.07	0.02	−0.02	−0.02	−0.02
		fusiform gyrus	37	L	−60	−56	−4	4.63	0.16	0.09	0.13	0.02	−0.03	0.01	−0.01

R1–7: RS contrast estimates.

**Table 6:** RS effect for sighted participants (coordinates in MNI space).

Cluster	Voxels	Region	BA	H	x	y	z	T	R1	R2	R3	R4	R5	R6	R7
1	44	posterior superior temporal gyrus	22	L	−60	−44	16	4.74	0.33	0.31	0.17	0.21	0.09	0.14	0.11
2	34	posterior superior temporal gyrus	22	R	68	−28	4	4.07	0.42	0.35	0.22	0.27	0.22	0.21	0.20
		posterior middle temporal gyrus	21	R	48	−28	−4	3.77	0.19	0.17	0.10	0.11	0.08	0.07	0.02
		posterior middle temporal gyrus	21	R	60	−28	−4	3.68	0.26	0.19	0.17	0.18	0.13	0.07	0.07

R1–7: RS contrast estimates.

Exclusive masking was used to identify voxels for which RS effects were not shared between the two groups. This analysis confirms stronger RS effect in the occipital and parietal cortices in the blind than in the sighted (see Figure 3B). However, the left fusiform gyrus did not survive this masking procedure. Finally, no voxels survived the inverse masking (RS effects in the control subjects masked by RS effects in the blind subjects) at the same threshold.

## Discussion

In the present study, CB and SI participants listened passively to short, repeated vowel sounds. The auditory stimulation resulted in bilateral activation in the transverse temporal and superior temporal gyri for both groups consistent with speech processing. Compared to SI adults and consistent with previous studies on auditory and speech and language processing in the blind [1],[4]–[5],[9] passive vowel processing activated bilateral primary and associative extrastriate visual cortex in the CB participants but not in the SI.

Our main purpose, however, was to investigate the presence and extent of RS effects in the CB during passive listening. Repetition suppression effects were observed as a linear BOLD signal decrease across the 7 consecutive vowels in auditory processing regions along the posterior part of the superior and middle temporal gyri for both groups overlapping in the right but only present in the left hemisphere for SI. Previous studies in sighted participants have consistently shown RS sensitivity of similar posterior auditory brain areas classically involved in speech and phonological processing [32]–[33]. For blind participants, however, a more extensive distribution of RS effects was found. The expanded regions of suppression were observed in extrastriate regions including the left fusiform gyrus and bilateral intraparietal sulcus (IPS) and supramarginal gyrus (SMG), the latter area associated with phonological processing [34] and visual word recognition [35]–[36] in SI and Braille reading in the blind [6]. The IPS, on the other hand, is involved in cross-modal interactions in SI including cross-modal links in attention [37].

For the CB, enhanced performance relative to SI has been reported for a wide range of behaviours from the ability to recognize rapid speech [13]–[14], [16]–[17] to detecting pitch change direction [11] to enhanced tactile acuity [38]–[40]. From these results, one possibility is that cross-modal plasticity in the CB is associated with more sensitive and/or efficient processing of sensory signals, including speech, in CB. The more extensive RS effects in the CB compared to the SI are consistent with this interpretation. However, RS effects have been shown to be sensitive to attention, which can also enhance or accentuate perceptual expectation (prediction) yielding neural response attenuation [23]. In the current study, we did not control for attention but assume that attentional factors, given the task, were minimal. In addition, attributing the RS effects to attentional differences in the two groups is difficult to support given the known differences in behavioral performance in a range of auditory based tasks in the CB compared to SI (see above). What can be suggested is that the RS differences during passive listening reflect an enhanced (more spatially extensive) obligatory predictive coding of sensory (auditory) input and cortico-cortical feedback [24]–[25], [27] in the CB. The enhanced neural processing would increase the sensitivity of the activated neurons by increasing the dynamic range, preventing saturation and increasing information encoding efficiency [41].

Whether the RS results are more directly attributable to differences in sensory processing or attention or basic mechanisms that underlie both (predictive coding), it is clear that the response of the CB participants differed from their sighted controls. Assuming that the current RS effects are representative of other auditory tasks for which CB perform better than SI, the current results suggest that cortical processing in CB may be optimized for auditory features, speech or otherwise. Moreover, enhanced RS effects or predictive coding may be a neural property differentiating processing in multiple cortical regions in the CB relative to SI. However, it should also be noted that previous reports of enhanced performance of the CB have been based on actual performance. Since the present study used passive listening only drawing a direct connection between the enhanced RS effects and behavior is tenuous.

Interestingly, activation of the parietal cortex, especially in the area of the IPS, suggests that unimodal sensory input in the CB activates multimodal cortex. It has been suggested that the sensitivity of multisensory heteromodal cortex like IPS is modulated by back-projection from sensory cortices [37]. In the context of speech perception, for example, multimodal sensory input (auditory and visual) integrates on heteromodal cortex and then back projects to the sensory cortices to modulate sensitivity. It is the case that in the CB this kind of interaction and modulation in heteromodal cortex during speech processing does not come from two different sensory inputs (auditory and visual), but from the same input (auditory). The speech input activates both the temporal cortex and extrastriate cortex and both project through the auditory visual dorsal streams to the heteromodal parietal cortex. That is, cross-modal plasticity in the CB is used to support auditory and visual convergence in the absence of direct visual receptor input. Through bi-directional projections the visual and auditory areas, driven by auditory input, could reinforce the predictive coding of stimulus input characteristics as a means to enhance further processing efficiency.

In summary, using the repetition of predictable speech stimuli, we were able to observe cross-modal neural processing differences in the congenitally blind that have not been reported previously. The present results, coupled with findings in the literature of superior processing and increased perceptual sensitivity in CB, suggest that sensory deprivation from birth may be responsible for a cascade of compensastory effects engaging cross-modal integration in the absence of multimodal sensory input resulting in enhanced and optimized predictive coding of sensory input. This computational framework is then used to enhance sensory processing and increase sensitivity and capacity for encoding stimulus features in the CB.
